# Infrared Thermography Sensor for Temperature and Speed Measurement of Moving Material

**DOI:** 10.3390/s17051157

**Published:** 2017-05-18

**Authors:** Rubén Usamentiaga, Daniel Fernando García

**Affiliations:** Department of Computer Science and Engineering, University of Oviedo, Campus de Viesques, 33204 Gijón, Spain; dfgarcia@uniovi.es

**Keywords:** temperature monitoring sensor, motion compensation, infrared thermography

## Abstract

Infrared thermography offers significant advantages in monitoring the temperature of objects over time, but crucial aspects need to be addressed. Movements between the infrared camera and the inspected material seriously affect the accuracy of the calculated temperature. These movements can be the consequence of solid objects that are moved, molten metal poured, material on a conveyor belt, or just vibrations. This work proposes a solution for monitoring the temperature of material in these scenarios. In this work both real movements and vibrations are treated equally, proposing a unified solution for both problems. The three key steps of the proposed procedure are image rectification, motion estimation and motion compensation. Image rectification calculates a front-parallel projection of the image that simplifies the estimation and compensation of the movement. Motion estimation describes the movement using a mathematical model, and estimates the coefficients using robust methods adapted to infrared images. Motion is finally compensated for in order to produce the correct temperature time history of the monitored material regardless of the movement. The result is a robust sensor for temperature of moving material that can also be used to measure the speed of the material. Different experiments are carried out to validate the proposed method in laboratory and real environments. Results show excellent performance.

## 1. Introduction

Temperature is one of the most measured physical properties. It describes the average kinetic energy of the molecules and atoms that make up a substance. Temperature provides information about the internal energy of an object, thus measurement, monitoring and control are crucial in most industrial processes.

Many different types of temperature sensors have been developed [1]. However, the most common are based on four different technologies: mechanical, electrical, ultrasonic and infrared. Most mechanical sensors are based on the volume of a fluid that changes with temperature. Mercury and alcohol are commonly used, although mercury based sensors are not sold any more due the toxicity and potentially harmful effects from broken thermometers. Electrical sensors are mostly thermocouples or thermoresistors. Thermocouples contain a junction of two dissimilar metal wires where voltage varies with temperature. Thermoresistors are made of semiconductors where the resistance varies rapidly and predictably with temperature. Ultrasonic sensors generate an ultrasonic wave and measure temperature based on the variation in the speed of propagation. Infrared sensors are based on the infrared radiation emitted by objects, which is mainly a function of their temperature.

Temperature sensors based on infrared thermography have many advantages over other types of sensors [2]. Infrared sensors are non-contact, thus they do not intrude upon the measurement. Moreover, they can measure the temperature of extremely hot objects. These sensors are also very fast, producing temperature readings in microseconds. They can also be grouped in an array of sensors called a focal plane array, in which each sensor provides information about the radiation at a single point, combining to produce a 2D thermal image. These advantages make sensors based on infrared thermography extremely useful in many different applications, such as electrical inspection [3], mechanical inspection [4], non-destructive testing [5], building inspection [6], industrial processes monitoring [7], medicine [8], cultural heritage diagnostics [9] or even pest detection [10].

Monitoring temperature using 2D thermal images equipped with infrared cameras means measurements can be taken at different areas of the scene simultaneously [2]. Moreover, these devices are able to acquire images at very high frame rates. Consequently, the temperature time history in these areas can be recorded and analyzed. This approach is commonly used in many different applications. For example, in [11] the temperature time history of pig iron is monitored while it is being poured. Analyzing the temperature time history is especially important for non-destructive testing applications. In these applications, objects are thermally stimulated to induce contrast between regions of interest [5]. The temperature time history at each point describes the thermal propagation of the external stimulation. Subsurface anomalies produce thermal variations during heating or cooling which sound areas do not. Hence, the analysis of the temperature time history can be used to detect thermal contrast, i.e., defects.

Under controlled conditions where the position of the camera does not change relative to the monitored object, the analysis of the temperature time history at different positions consists of the selection of different pixels, or an area of pixels, in the image. The intensity value of these pixels, or the average intensity if an area is selected, is calculated from the sequence of images, providing the required temperature time history. However, in many different environments measurements are affected by vibrations [12], which can be described as periodic or random motion from an equilibrium position. Vibrations can affect the camera or the monitored object. In either case, the selected position in the image will not correspond to the same area of the monitored object in consecutive images, which invalidates the calculation of the temperature time history. A different scenario, but with similar consequences is when the monitored object or material is moving. Again, a static selection of points in the image cannot be used to calculate the temperature time history of the regions of interest.

The compensation for unwanted camera motion, generally called image stabilization has been widely studied with visible images [13,14,15]. However, in the case of infrared images research works is scarce, and generally focused on particular applications. In [16], the authors propose an image stabilization algorithm for infrared images based on the 2D Fourier Transform. In this case, the problem is focused on the analysis of the temperature distribution in biomedical applications, where motion appears because patients move due to breathing, pulse and other voluntary and involuntary reactions. In [17] a similar approach is applied to compensate for vibrations in online welding monitoring. In this case, a combination of point tracking and direct phase substitution is used. Both works assume that vibrations only provoke slight movements of the camera relative to the inspected object. Moreover, they do not consider the problem of temperature monitoring when the object is really moving, i.e., when the position of the region of interest in the image also changes because the object is moving, not just affected by vibrations.

Vibration control and compensation is an active research field with numerous developments [18,19]. Mechanical sensors include different components to compensate for vibrations. Generally, a sensor measures the vibrations using an accelerometer. Then, the resulting signal is transformed so that different actuators can generate movements that compensate for the detected vibrations. Digital sensors use the acquired images to extract features that can be used to detect the vibrations. Then, they transform subsequent images to compensate for the movements.

In this work, a general solution is proposed for the problem of temperature monitoring of moving material. The proposed procedure includes tracking. Therefore, not only the image stabilization problem is solved, but also the calculation of the temperature time history of moving material. Both vibrations and movement are treated equally. The solution to these problems is unified in a single method composed of three steps: image rectification, motion estimation and motion compensation. Image rectification calculates a transformed image with a front-parallel projection where measurements in real-world units (mm), rather than pixels, can be carried out. Assuming all points of interest lie on the same plane, then motion can be described accurately using a 2D rigid body transformation. This approach greatly simplifies the mathematical model used to describe motion and also the compensation. Motion is estimated using a combination of feature detection applied to the rectified images and robust model estimation. The proposed method is evaluated in different applications in laboratory experiments and also in real industrial environments.

The main contribution of this paper is the proposal of a new sensor for temperature of moving objects. The sensor is based on infrared thermography, and keeps track of the movements between the infrared camera and the material to calculate the temperature time history accurately. Processing infrared images is a challenging task because standard algorithms do not provide good results. Therefore, specific procedures are proposed. The proposed solution can also be applied to measurement scenarios where the camera or the inspected object are affected by vibrations. This work includes camera calibration, therefore, it produces the correct temperature time history using a simple yet accurate linear mathematical model. Moreover, the real speed of the material can be calculated at any point in time. The intelligent sensor proposed in this work can provide accurate readings regardless of the movement. Excellent performance is obtained in terms of accuracy and robustness.

Including the compensation for the movement of the monitored material using rectified images present a novel approach to solve the considered problem. It provides not only a very accurate method to model motion but also a robust method to measure the speed of the material. This provides a major advantage when designing a sensor that needs to provide accurate information about a signal, temperature in this case, of a material that is moving at variable speed. Moreover, it can also be used to detect when the material is moved or stopped, which can be key to detecting abnormal measurement patterns correctly.

The remainder of this paper is organized as follows. Section 2 presents the proposed approach for temperature monitoring; Section 3 discusses the results obtained with real data; and finally, Section 4 reports conclusions.

## 2. Monitoring Procedure

**Figure 1:**
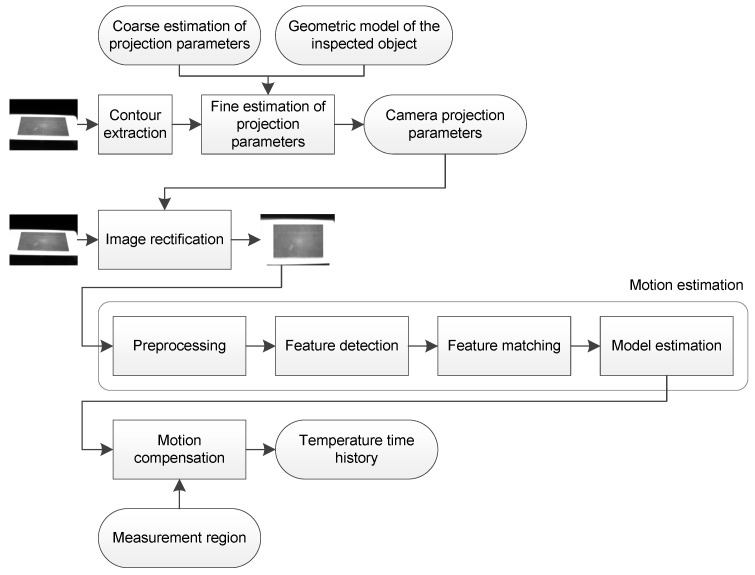
Summary of the proposed approach.

The temperature monitoring procedure proposed in this work first acquires the images using an infrared camera. Images are rectified to calculate a front-parallel projection, removing perspective distortion. This step requires the estimation of the camera projection parameters. The proposed method is based on the extraction of the contour of the inspected object and an iterative approximation to the reference shape. Next, motion estimation and compensation is applied to the rectified images. This requires a preprocessing procedure to enhance the contrast in the images. Features are extracted from these enhanced images and used to estimate the movement model robustly. Finally, the temperature time history of the inspected object is calculated. The following sections describe the details of these steps. Figure 1 shows a summary of the steps.

### 2.1. Image Acquisition

The first required step in order to monitor the temperature is the acquisition of the infrared images. The images are acquired using an infrared camera. These devices measure infrared radiation in a particular wavelength, typically from 8 to 12 μm, or from 2 to 5 μm. The measured infrared radiation is converted into temperature based on the properties of the inspected material, including the emissivity, and the conditions in which the image was acquired, including reflected temperature, ambient temperature, distance, or relative humidity. The accuracy of the calculated temperature values is greatly affected by errors in the estimation of these parameters.

The most common cameras used to acquire infrared images in industrial applications are long-wavelength infrared cameras based on uncooled microbolometers that operate in the range from 8 to 12 μm. They do not require cooling. However, the acquisition rate is low compared with high-end mid-wavelength infrared cameras that operate in the range from 2 to 5 μm. These cameras are usually based on cooled semiconductor detectors that provide much better temperature resolution and higher speed, but they are also more expensive and require more maintenance. Thus, both camera types have their advantages and disadvantages. In the case of fast moving objects, the proposed procedure would require high-end cameras to operate correctly and avoid blurred images. However, the proposed monitoring procedure can be applied using any type of camera.

Vibrations or camera motion cause not only a shifting of the monitored object in the image, they can also cause blurring of the image. Cooled cameras require short integration times (around 1 to 1.5 ms at room temperature). However, a microbolometer detector usually requires an integration time ten times higher. Therefore, depending on the speed of the movement between the camera and monitored object, motion blurring could appear in the acquired images. This work does not deal with this issue. It is assumed that moving objects are exposed sharply and edges can be detected accurately, either because objects move slowly or because a high-end camera based on cooled semiconductor detectors is used. In case motion blurring cannot be avoided, motion deblurring should be applied to the images before applying the proposed procedure. This issue is studied with detail in [20] for visual images. Reference [21] proposes a procedure for motion deblurring of infrared images from a microbolometer camera.

### 2.2. Image Rectification

In this work image rectification is used to calculate a front-parallel projection of the images that can be used to estimate the motion between images accurately. Image rectification requires an estimation of the parameters that control camera projection. These parameters can be classified as extrinsic or intrinsic camera parameters.

Extrinsic parameters control the transformation of points in world coordinates to points in camera coordinates, and include three rotations and three translations. This transformation can be expressed as (1).

(1) $$T_{ext}=\left(\begin{array}{cccc} r_{11} & r_{12} & r_{13} & t_{x}\\ r_{21} & r_{22} & r_{23} & t_{y}\\ r_{31} & r_{32} & r_{33} & t_{z}\\ 0 & 0 & 0 & 1 \end{array}\right)$$

Intrinsic parameters determine the projection of points from camera coordinates to pixels in the image, and include the focal length (*f*), the size of the pixels (width and height: $S_{x}$ and $S_{y}$), and the position of the central pixel ($C_{x}$ and $C_{y}$). This projection can be described as the combination of a perspective projection from 3D to 2D, expressed as (2), and a 2D affine transformation, expressed as (3).

(2) $$T_{proj}=\left(\begin{array}{cccc} f & 0 & 0 & 0\\ 0 & f & 0 & 0\\ 0 & 0 & 1 & 0 \end{array}\right)$$

(3) $$T_{aff}=\left(\begin{array}{ccc} \frac{1}{S_{x}} & 0 & C_{x}\\ 0 & \frac{1}{S_{y}} & C_{y}\\ 0 & 0 & 1 \end{array}\right)$$

The transformation of a 3D point $P={\left(x^{w},y^{w},z^{w}\right)}^{T}$ in world coordinates into a 2D point $p={\left(r,c\right)}^{T}$ in pixel coordinates can be expressed as (4), where *r* and *c* stand for pixel row and column in the image.

(4) $$\begin{array}{cc} p & =T_{aff}T_{proj}T_{ext}P\\ \left(\begin{array}{c} r\\ c \end{array}\right) & =\left(\begin{array}{ccc} \frac{1}{S_{x}} & 0 & C_{x}\\ 0 & \frac{1}{S_{y}} & C_{y}\\ 0 & 0 & 1 \end{array}\right)\left(\begin{array}{cccc} f & 0 & 0 & 0\\ 0 & f & 0 & 0\\ 0 & 0 & 1 & 0 \end{array}\right)\left(\begin{array}{cccc} r_{11} & r_{12} & r_{13} & t_{x}\\ r_{21} & r_{22} & r_{23} & t_{y}\\ r_{31} & r_{32} & r_{33} & t_{z}\\ 0 & 0 & 0 & 1 \end{array}\right)\left(\begin{array}{c} x^{w}\\ y^{w}\\ z^{w}\\ 1 \end{array}\right) \end{array}$$

In order to estimate the optimal values for the projection camera parameters, observations of a known target are required. Feature extraction from the images provides the position of known reference points in the calibration target. The parameters of the camera projection model are then estimated by using direct or iterative methods based on this set of reference points. This approach for the estimation of the camera parameters requires calibration targets with features of known dimensions. In visible cameras, accurate calibration targets can be accurately printed using off-the-shelf printers. However, infrared cameras require calibration targets with distinguishable features in terms of infrared radiation.

A recent work on infrared camera calibration estimates the projection parameters in (4) without using specific calibration targets [22]. In this work the projection parameters are estimated with iterative approximations based on the position of the edges in the image, which represent a great advantage for infrared image because objects of interest in infrared images can be easily distinguished from the environment due to temperature differences. This procedure does not consider distortions, but provides accuracy acceptable for common infrared applications. This method is easy to apply, and it does not require specific calibration targets because it is based on information that can be extracted from objects in the image. Moreover, it can be applied from only one image of a known object. Therefore, this is the method used to estimate camera projection parameters in this work.

The considered rectification procedure assumes that the area where the measurement is performed is flat. Therefore, the extracted points from the images lie on the same plane. This plane, the measurement plane, appears in many different applications where infrared thermography is used, such as building inspection or non-destructive testing where the inspected specimens are usually flat. Considering this plane Z=0 then all world points have a $z^{w}$ equal to zero. Thus, (4) can be expressed as (5).

(5) $$\begin{array}{cc} \left(\begin{array}{c} c\\ r\\ 1 \end{array}\right) & =\left(\begin{array}{ccc} \frac{f}{S_{x}} & 0 & C_{x}\\ 0 & \frac{f}{S_{y}} & C_{y}\\ 0 & 0 & 0 \end{array}\right)\left(\begin{array}{ccc} r_{11} & r_{12} & t_{x}\\ r_{21} & r_{22} & t_{y}\\ r_{31} & r_{32} & t_{z} \end{array}\right)\left(\begin{array}{c} x^{w}\\ y^{w}\\ 1 \end{array}\right)\\ \left(\begin{array}{c} c\\ r\\ 1 \end{array}\right) & =H\left(\begin{array}{c} x^{w}\\ y^{w}\\ 1 \end{array}\right) \end{array}$$

The iterative method proposed to estimate the coefficients of *H* requires a coarse estimation of the coefficients. The initial coarse estimation of the intrinsic parameters is provided by the manufacturer of the particular camera used (focal length, detector pitch and IR resolution). The initial estimation of the extrinsic parameters consists of the estimation of the displacement (vertical, horizontal and distance) and rotation (pan, tilt and roll) of the measurement plane relative to the camera.

The estimation of the projection parameters continues from the coarse estimation of *H*. A contour of the inspected object is extracted from the image and transformed into world coordinates using $H^{-1}$. The proposed method to extract the contour of the inspected object is the Canny edge detector [23]. Then, correspondences are estimated by computing the closest points from the model to the object after applying the transformation to world coordinates. Incorrect correspondences bias the procedure, thus they must be filtered using robust statistics. The final step is the estimation of a homography using the correspondences. The procedure is repeated until convergence is reached. In each iteration the distance from the extracted contour to the real shape of the object is reduced. The result is a homography that describes the projection parameters accurately. This transformation can be directly applied to the original infrared image in order to obtain the rectified image in world coordinates.

**Figure 2:**
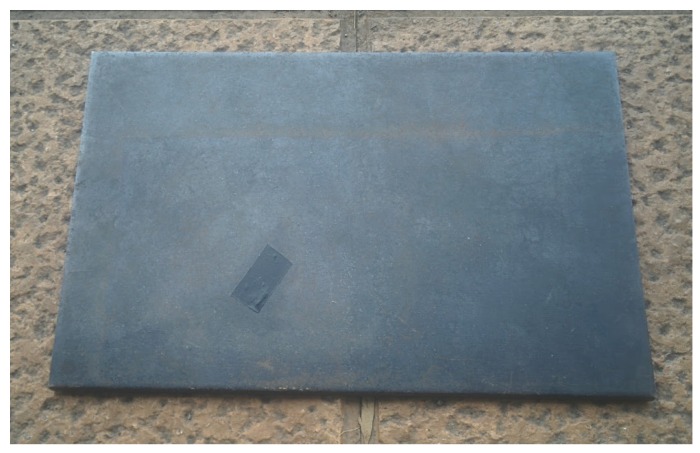
Visible spectrum image of the test piece.

In order to illustrate this procedure, a solid object is manually moved while temperature monitoring is performed. The goal is to measure the temperature in a particular location regardless of the movement. This experiment simulates the temperature monitoring of material that is moved, for example hot metal stones on a conveyor belt, or affected by vibrations. Next section will extend the tests with images acquired in real environments. In this example, a test piece made up of metal is used. The dimensions of the test piece are 300 mm × 199 mm × 5 mm. A visible image of the test piece can be seen in Figure 2. The test piece is placed on a hot plate (electric griddle), which is at 150 ${}^{\circ }$C approximately. The experiment is performed when the test piece is around 100 ${}^{\circ }$C.

**Figure 3:**
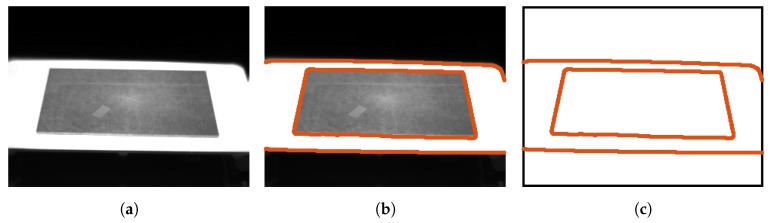
Test piece used to illustrate the rectification procedure. (**a**) Infrared image of the heated test piece; (**b**) Edges in the image; (**c**) Extracted contour of the objects in the image.

An infrared image of the test piece placed on the hot plate can be seen in Figure 3a. This image is the first of a sequence of images acquired while the test piece is moved within the measurement plane to simulate movements of the material or vibrations. A piece of electrical tape is stuck on the surface for later tests. The temperature of the electrical tape is nearly identical to the underlying test piece and it is used for temperature monitoring. The electrical tape can be clearly distinguished in the images because the emissivity of the tape is higher than the emissivity of the surface of the metal test piece. Therefore, at the same temperature it emits more infrared radiation.

In order to extract the contour of the test piece, an edge detector is applied to the image. The result can be seen in Figure 3b. The extracted contour in Figure 3c is used for the estimation of the projection parameters.

The infrared camera used in this experiment is a FLIR T450sc (FLIR Systems, Wilsonville, OR, USA). The manufacturer provides information that can be used to obtain a coarse estimation of the projection parameters: 18 mm focal length, 25 μm detector pitch and 320 × 240 image resolution. For the initial values of the extrinsic parameters the following values are roughly estimated: 3${}^{\circ }$ pan, 54${}^{\circ }$ tilt, 180 mm and 180 mm horizontal and vertical displacements, and 1200 mm distance. This camera can acquire raw infrared images, and lossless videos. Therefore, the images used in the tests are not corrupted by noise, for example due to JPEG compression. The technical specifications of this camera are given in Table 1.

**Figure 4:**
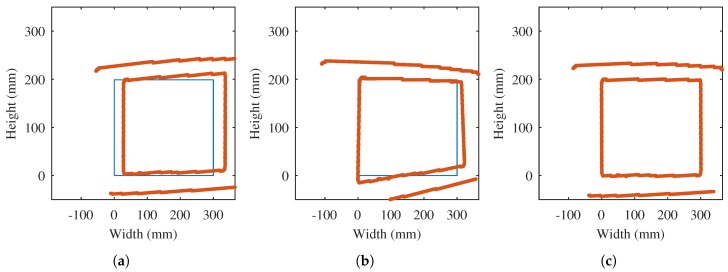
Iterative estimation of the projection parameters. (**a**) Initial estimation; (**b**) Iteration 5; (**c**) Final iteration.

The initial values of the projection parameters are used to transform the extracted contour to world coordinates. The result can be seen in Figure 4a. The shape of the inspected object (test piece is 300 mm × 199 mm) is included in the figure. As can be seen, the transformation of the extracted contour does not match the real shape of the object, as it is only an approximation.

The fine estimation of the projection parameters is carried out by minimizing the distance from the extracted contour in world units to the real shape of the object. The procedure runs iteratively until convergence. In each iteration the approximation improves, that is, the estimation of the projection parameters is more accurate. Figure 4b shows the results after 5 iterations. Figure 4c shows the results when convergence is reached, where an accurate estimation of the projection parameters is obtained.

Not all the points in the extracted contour produce valid correspondences. As can be seen in Figure 4, the edges of the base plate are not part of the shape of the test piece. Therefore, these points are discarded [24].

**Figure 5:**
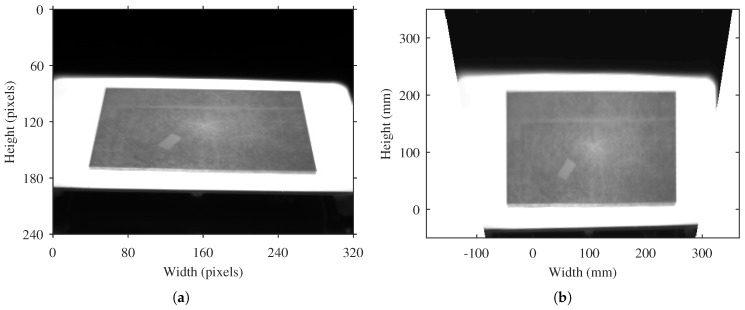
Result of the image rectification procedure. (**a**) Original image; (**b**) Rectified image.

The final valid correspondences are used to accurately estimate the projection parameters, that is, the homography that describes the projection of points from world coordinates to image coordinates, and vice versa. The estimated homography is used to rectify the infrared image. In this procedure the image is interpolated according to a rectangular grid in order to calculate a front-parallel projection of the image using the projection parameters described in the homography. This procedure is generally available in most image processing packages as a projective transformation of an image [25]. The result can be seen in Figure 5, where an image with pixel coordinates is transformed into an image with a front-parallel projection in real-world units. The coordinates of the image in Figure 5b are real-world units. Thus, useful geometric information can be easily extracted from the image.

### 2.3. Motion Estimation

Motion estimation is required to compensate for the movement of the monitored material in the sequence of images. This movement must be described with a mathematical model. Therefore, motion estimation requires the estimation of the values of the mathematical model. Once the model is estimated, it can be used to compensate for the movement of the object, by applying the inverse transformation to the objects that have moved in the image.

#### 2.3.1. Mathematical Model

Modeling the movement between two images is complex, as an image provides a 2D representation of a 3D scene. However, rectified images provide a major advantage in this aspect as pixels represent world coordinates in the measurement plane. This way, the mathematical model required to describe movement is greatly simplified, yet accurate and complete. In a rectified image, the movements of the objects is 2D. Thus, it can be modeled using a 2D rigid transformation. This transformation has three coefficients: the rotation angle θ; and the horizontal and vertical translations: tx and ty. This transformation can be expressed as (6).

(6) $$M=\left(\begin{array}{ccc} \cos(\theta ) & -\sin(\theta ) & tx\\ \sin(\theta ) & \cos(\theta ) & ty\\ 0 & 0 & 1 \end{array}\right)$$

Working with rectified images has many advantages. One of them is that the model of the movement is very simple.

#### 2.3.2. Feature Detection

This step detects salient and distinctive features from the images. Features must be distributed over the image. Also, the same features must be efficiently detectable in consecutive images. The goal is to find matching features between consecutive images, that is, a feature that identifies the same point in the scene in the two images. Generally, these features are detected from distinctive locations in the images, such as region corners or line intersections.

Feature detection includes two parts: the detection of the points of interest, and the description of these points. Points of interest in the image are stable and repeatable positions in the image. In visible images, these points can correspond to corners. A vector of features is calculated then for each of these points. These features include derivatives, or moment invariants. One of the most used method for feature detection is SURF (Speeded Up Robust Features) [26]. This method is based on Hessian detectors and use the Haar wavelet to calculate the features of the detected points.

SURF does not provide good results using raw infrared images because in most cases the contrast in the region of interest is not enough to detect the features required to estimate movement. Moreover, when the image contains information about the moving material but also about non-moving objects, such as the background, features can also be detected in non-moving areas. This mixture of features cannot be used to estimate movement. Therefore, a preprocessing stage is proposed.

The first step of the preprocessing stage is to extract the region of interest from the image, that is, the part where the moving material is located. This step is application dependent, but can be carried out in most cases using thresholding techniques [27]. The moving material inspected using infrared thermography usually has a different temperature from the rest of the image. Thus, thresholding the image based on the temperature level is an effective solution that works for most applications. The example presented in Figure 3 is slightly different because in the image three parts can be distinguished based on temperature: the background, the hot plate and the test piece. In this case an effective approach is to apply thresholding twice: a first thresholding to distinguish the plate and the test piece from the background, and then a second thresholding applied only to the extracted region in the first thresholding to distinguish the plate from the test piece.

The second step of the preprocessing stage is the enhancement of the contrast in the image. This step enables SURF to extract meaningful features from the region of interest in the image. Applying SURF to the raw image can result in a low number of features focused on the corners of the material that do not provide the required information to estimate movement correctly. One of the most common methods to enhance contrast in images is a method known as CLAHE (Contrast Limited Adaptive Histogram Equalization) [28]. This is the proposed method for contrast enhancement in this work.

**Figure 6:**
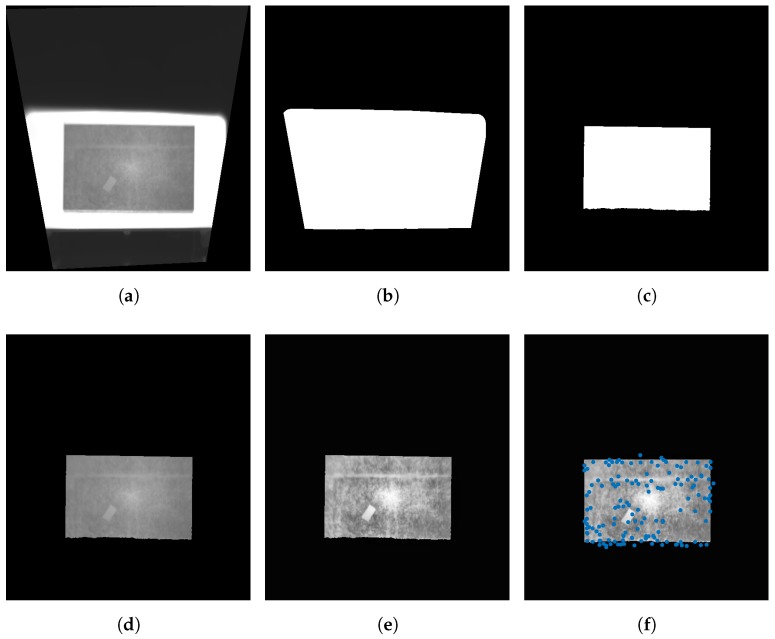
Result of the feature detection procedure. (**a**) Rectified image; (**b**) Region extracted after the first thresholding; (**c**) Region extracted after the second thresholding; (**d**) Extracted object of interest; (**e**) Contrast enhancement of the image for the object of interest; (**f**) Location of the detected features.

Figure 6 shows the results of the feature detection procedure for the test piece used in the previous example. Figure 6b shows the results of the first thresholding, where a region that includes the hot plate and the test piece is obtained. This first thresholding is applied to distinguish the hot plate and the test piece from the background. The result is a binary image, where the white part represents the foreground and the black part the background that is ignored in next steps. The results of the second thresholding are shown in Figure 6c. In this case, the obtained regions distinguish the test piece from the plate. The white area in this image represent the region of interest for the considered problem: the region in the image where the test piece is located. Using this region in the original image in Figure 6a produces the result shown in Figure 6d. This image is obtained by multiplying the images in Figure 6a,c (in some references this is described as the application of the and logical operator to the images). Figure 6e shows the result of the next step in the preprocessing: contrast enhancement using CLAHE. The resulting image can now be used to detect the features required to estimate the movement. The location of the features for the example can be seen in Figure 6f.

As can be seen in Figure 6f, some features are located outside the boundary of the test piece. This is because features are calculated based on derivatives that use windows of pixels around the pixel in which the derivative is calculated. Cropping the image around the test piece would solve this problem, but some interesting features in the corners could be missed.

#### 2.3.3. Feature Matching

Feature matching looks for correspondences between two set of features. Features from the two considered images are compared and linked by minimizing the sum of squared differences. The result is a set of possible correspondences, in most cases containing outliers.

**Figure 7:**
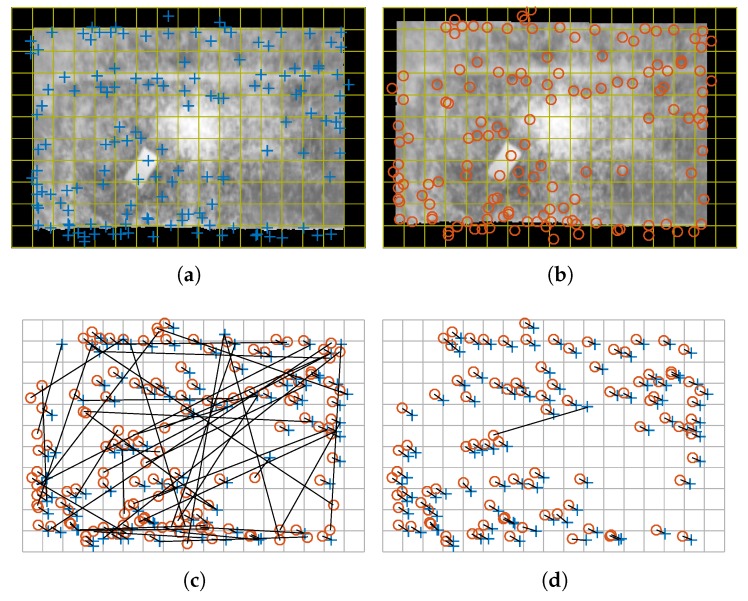
Result of the feature matching procedure. (**a**) Features from the first image; (**b**) Features from the second image; (**c**) Results of the feature matching; (**d**) Results of the feature matching using heuristics.

Figure 7 shows an example of feature matching. In this example a second image of the same test piece acquired later is shown. When the second image is acquired the test piece is slightly moved to the left and upwards. The movement of the test piece is performed within the measurement plane, thus, the same projection parameters are used to rectify the second image. The feature detection procedure is applied to the two images, including preprocessing and enhancement. The results are shown in Figure 7a,b. The goal of the feature matching procedure is to find the corresponding features between the two images. The initial result of the feature matching procedure can be seen in Figure 7c. A line connects the matched features between the two images. They also indicate the estimated movement, from the crosses to the circles. The initial result includes many outliers that do not provide the correct information about the movement of the test piece. Ideally, all the matched features should identify the same movement. Part of these outliers can be removed using heuristics. For example, distances between feature vectors can be sorted. Then, only a percentage of the closest distances can be selected as valid in order to reject ambiguous matches. Multiple features in the first image matching the same feature in the second image can also be removed to reduce the number of outliers. Using these two heuristics, the result of the feature matching procedure reduces the number of outliers, as can be seen in Figure 7d. However, no heuristic can guarantee there will not be outliers in the result of the matching. In the example, there are still clearly visible outliers.

When using infrared images, features can also change with time due to temperature differences that can diminish due to heat diffusion. Therefore it is not possible to find matching features between images acquired at distant time periods. In this work feature matching is applied between images acquired consecutively, where the features are expected to remain constant. However, temperature differences could generate some outliers that need to be considered for the model estimation.

#### 2.3.4. Model Estimation

The movement model is described using a 2D rigid transformation. The coefficients of this model must be estimated using the result of the feature matching procedure: a set of point correspondences. These correspondences provide information about the movement of the material in the image. In this work, the method used to estimate a rigid transformation is a fast 2D method [29].

Considering a set of *n* points $P=\{p_{1},p_{2},\ldots ,p_{n}\},$ and $Q=\{q_{1},q_{2},\ldots ,q_{n}\}$ in $R^{2}$, where $p_{i}={\left(p_{ix},p_{iy}\right)}^{T}$ and $q_{i}={\left(q_{ix},q_{iy}\right)}^{T}$ represents the 2D coordinates of the *i*-th point in P and Q, the rigid transformation that maps P into Q can be described as (7), where *R* is the rotation and *t* the translation.

(7) Q=PR+t

Solving (7) requires minimizing *E*, which is obtained using the least squares error criterion and can be defined as (8).

(8) $$E=\sum_{i=1}^{n}{\left|Q-PR-t\right|}^{2}$$

The value of *t* that minimizes *E* must satisfy (9).

(9) $$0=\frac{\partial E}{\partial t}=-2\sum_{i=1}^{n}\left|Q-PR-t\right|$$

Therefore, *t* can be calculated using (10), where $\bar{p}$ and $\bar{q}$ are the centroids of P and Q

(10) $$t=\bar{q}-R\bar{p}$$

Substituting the centered points $P^{z}=\left\{p_{1}^{z}=p_{1}-\bar{p},p_{2}^{z}=p_{2}-\bar{p},\ldots ,p_{n}^{z}=p_{n}-\bar{p}\right\},$ and $Q^{z}=\left\{q_{1}^{z}=q_{1}-\bar{q},q_{2}^{z}=q_{2}-\bar{q},\ldots ,q_{n}^{z}=q_{n}-\bar{q}\right\}$ in (8) yields (11).

(11) $$E=\sum_{i=1}^{n}{\left|Q^{z}-P^{z}R\right|}^{2}$$

The angle of rotation θ defines the rotation matrix. The rotation of point $p_{i}^{z}$ using this angle is (12).

(12) $$Rp_{i}^{z}=\left(\begin{array}{c} \cos\left(\theta \right)p_{ix}^{z}-\sin\left(\theta \right)p_{iy}^{z}\\ \sin\left(\theta \right)p_{ix}^{z}+\cos\left(\theta \right)p_{iy}^{z} \end{array}\right)$$

Substituting (12) in (11) gives an equation where *E* only depends on θ. Solving for θ results in (13).

(13) $$\theta =\tan^{-1}\left(\frac{\sum_{i=1}^{n}\left(p_{ix}^{z}q_{iy}^{z}-p_{iy}^{z}q_{ix}^{z}\right)}{\sum_{i=1}^{n}\left(p_{ix}^{z}q_{ix}^{z}+p_{iy}^{z}q_{iy}^{z}\right)}\right)$$

In order to calculate the translation *t*, the value of *R* must be substituted in (10).

The method used to estimate the rigid transformation between correspondences should only be applied when there are no outliers in the data. Correspondence outliers would lead to major errors in the resulting estimated transformation. Therefore, the method to estimate the rigid transformation cannot be applied to the matched features directly.

The proposed solution for the estimation of the rigid transformation using noisy correspondences is MLESAC [30]. This robust estimator is an enhanced version of the Random Sample Consensus (RANSAC) algorithm [31], widely applied to estimate mathematical models robustly. The algorithm randomly samples the available correspondences and estimates rigid transformations using the previously described method. Not all point correspondences are used, just the strictly required number to estimate the rigid transformation. Among all the putative solutions, the solution that maximizes the likelihood is chosen.

**Figure 8:**
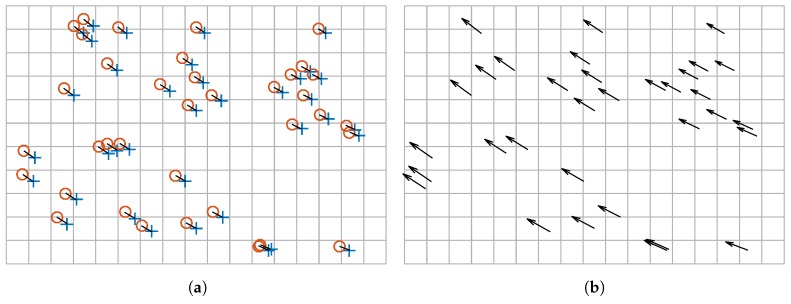
Result of the motion estimation procedure. (**a**) Results of the feature matching after the robust model estimation; (**b**) Estimation of the movement (arrows are not to scale).

Figure 8 shows the results of the motion estimation procedure for the considered example. As can be seen in Figure 8a, only some of the correspondences in Figure 7d are truly considered for the robust estimation of the movement model. The final result represented in Figure 8b is an accurate estimation of the movement in the test piece between the two images. The result of the robust estimation of the movement is a 2D rigid transformation that perfectly describes the movement of the material in the measurement plane.

### 2.4. Motion Compensation

Motion compensation can be applied in two equivalent ways. The first possible approach is to move the pixels in the second image according to the inverse of the estimated movement. This approach requires image reinterpolation.

**Figure 9:**
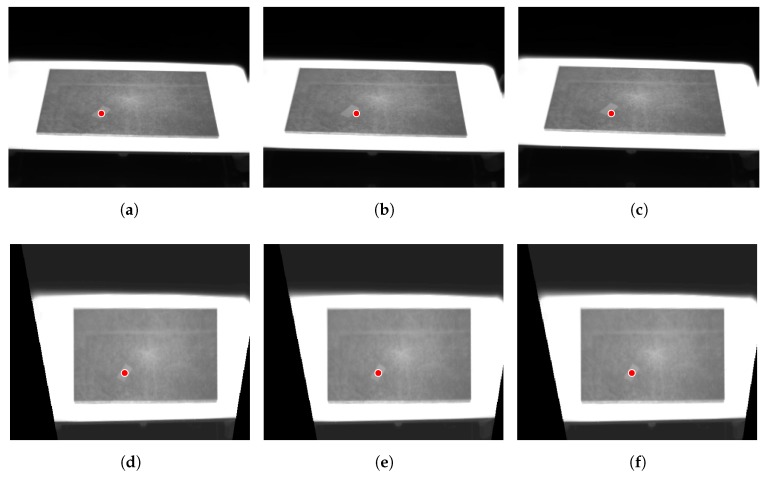
Result of the motion compensation procedure. (**a**) Raw image at $t_{0}$; (**b**) Raw image at $t_{1}$; (**c**) Raw image at $t_{2}$; (**d**) Motion compensated image at $t_{0}$; (**e**) Motion compensated image at $t_{1}$; (**f**) Motion compensated image at $t_{2}$.

Figure 9 shows an illustration of the image reinterpolation approach. The first row of images shows the raw infrared images acquired while the test piece is moved. In the first image ( Figure 9a), a circular measurement region is established on the electrical tape stuck on the test piece. As expected, while the test piece is moved the position of the measurement region misses the location of the center of the tape. The second row in the figure shows the images after motion compensation. In this case, the circular measurement region always stays at the same position relative to the electrical tape, regardless of the movement.

**Figure 10:**
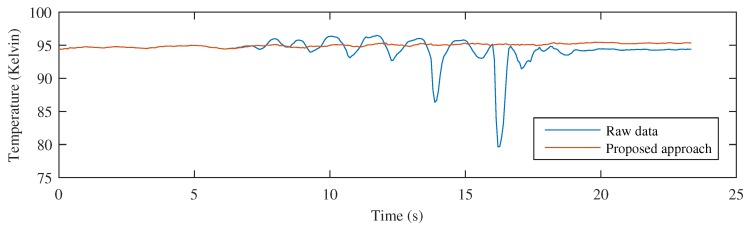
Comparing temperature monitoring using raw images and the proposed approach.

Monitoring the temperature in the circular measurement region of the previous example provides the results shown in Figure 10. When the raw images are used, the position of the circular measurement region fails to identify the position of the center of the tape, as can be seen in Figure 9. Therefore, the resulting signal does not provide the correct temperature of the tape over time. However, when the movement is compensated using the proposed approach, the position of the measurement region is always correct, resulting is an accurate signal representing the temperature time history of the inspected material.

The second approach to image reinterpolation is to move the measurement regions according to the estimated movement of the monitored material. This approach does not require image reinterpolation, thus, it is faster and produces the same results.

The motion estimation procedure produces a 2D rigid transformation $H_{i}$ between every two consecutively acquired images, $I_{i-1}$ and $I_{i}$. The obtained transformations can be composed to obtain the transformation from the first image to the current image *i* using (14).

(14) $$H_{T,i}=\prod_{j=0}^{j=i}H_{j}$$

Using (14) any single point in one image can be transformed back and forth between any other image. Therefore, it can be used to compensate for the movement of the material.

## 3. Results and Discussion

In order to test the proposed procedure, a first experiment is performed with the same test piece with a different orientation and movement. The test piece is placed on a hot plate. The goal is to monitor the temperature of a piece of electrical tape stuck on the test piece while it is moved, simulating vibrations.

**Figure 11:**
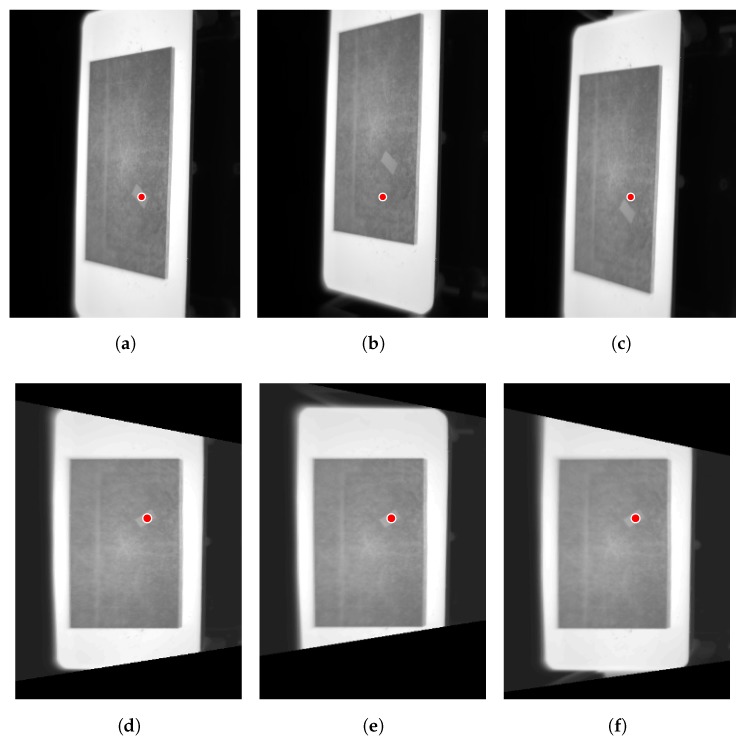
Result of the motion compensation procedure for the test piece with different orientation and movement. (**a**) Raw image at $t_{0}$; (**b**) Raw image at $t_{1}$; (**c**) Raw image at $t_{2}$; (**d**) Motion compensated image at $t_{0}$; (**e**) Motion compensated image at $t_{1}$; (**f**) Motion compensated image at $t_{2}$.

The results of the experiment can be seen in Figure 11. The first step is the image rectification using the estimated projection parameters. The results of this procedure are similar to those described for the test piece in the original orientation. Using the rectified images, motion is estimated and compensated. Images are reinterpolated in order to compensate for the movement. In this experiment, the movement of the test piece is increased, as can be seen in the figure. However, this does not affect the estimation and compensation of the movement, producing accurate results. Therefore, robust temperature monitoring can be performed regardless of the movement. As can be seen, the measurement region is always in the same position relative to the test piece.

**Figure 12:**
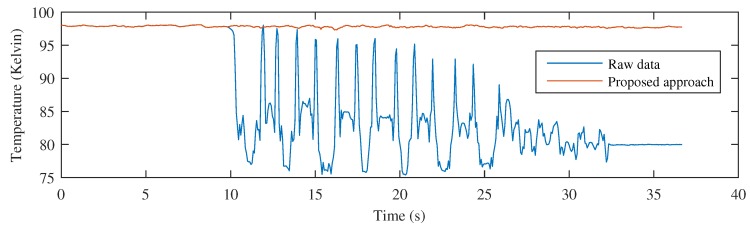
Comparing temperature monitoring using raw images and the proposed approach for the test piece with different orientation and movement.

The monitored temperature can be seen in Figure 12. The resulting temperature signal when using the proposed approach provides the correct information about the temperature in the region of interest. This result can be compared with the temperature signal obtained from the raw images. In this case, the temperature time history is incorrect because it is affected by the movement of the material.

In order to calculate the temperature of the test piece in the experiments, the infrared camera was configured using the emissivity of the electrical tape: 0.96 (Scotch^TM^ Premium Vinyl Electrical Tape 88, 3M, Maplewood, MN, USA); the reflected temperature estimated using the reflector method [32]: 22.4 ${}^{\circ }$C; and the distance, ambient temperature and relative humidity.

Experiments have also been performed in a real environment: a sinter cooler. Sinter is a solidified porous material used in the steel industry. It is created by applying heat and pressure to a mixture of different raw materials including fine particles of iron, and other materials such as limestone and coke [33]. The material is later moved to a rotatory cooler where the temperature must be monitored before the final transportation using a conveyor belt to the next step of the industrial process in the blast furnace, where pig iron is produced.

**Figure 13:**
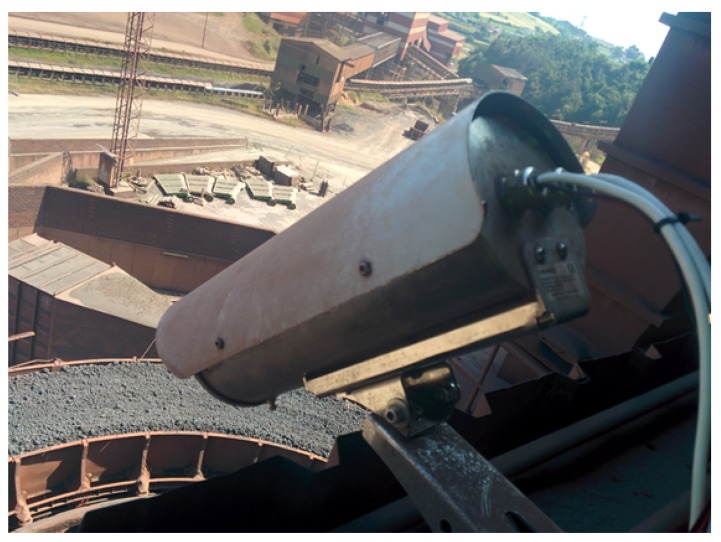
Infrared camera for sinter monitoring.

The sinter cooler is a 3.2 m wide circular rotatory ring where air is blown from fans. The cooler moves slowly while the sinter material cools. Temperature monitoring in the cooler is critical to ensure that the cooling pattern is correct. Moreover, temperature monitoring is also used to prevent fires due to excessive temperature in the transportation by conveyor belt. Figure 13 shows an image of the rotatory cooler and the camera used for monitoring.

**Figure 14:**
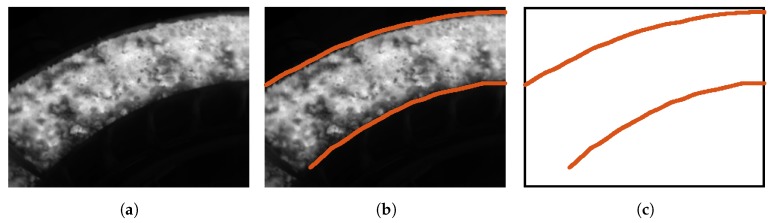
Result of the contour extraction for the sinter material. (**a**) Infrared image of the sinter in the cooler; (**b**) Edges in the image; (**c**) Extracted contour of the cooler in the image.

Figure 14 shows an infrared image of the cooler and the contour extraction procedure. Due to the optics of the camera and the distance from the camera to the object, only a partial view of the cooler is available. However, this visible part is enough to apply the proposed rectification procedure. The first step is to extract the contour of the cooler. This can be performed by applying an edge detector to the image. The sinter material is hotter than the rest of the image. Thus, it can be clearly distinguished. The extracted contour is the boundary of the visible part of the cooler in the image.

In this example a FLIR A315 infrared camera (FLIR Systems, Wilsonville, OR, USA) is used. The information provided by the manufacturer and an estimation of the pan, tilt and distance to the cooler is used for the coarse estimation of the projection parameters. The technical specifications of this camera are given in Table 2.

**Figure 15:**
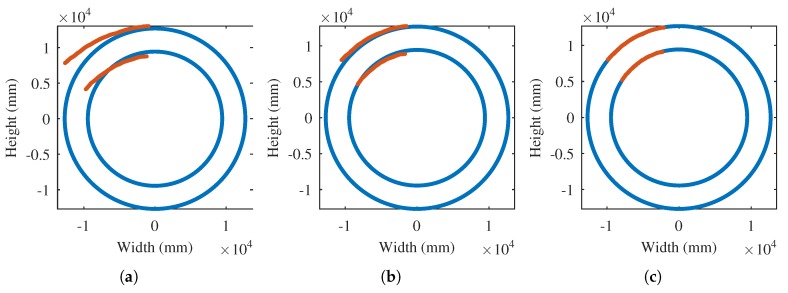
Iterative estimation of the projection parameters for the sinter material. (**a**) Initial estimation; (**b**) Iteration 10; (**c**) Final iteration.

Figure 15a shows the transformation of the extracted contour to world coordinates. In this same figure, a model of the cooler ring is represented. The information about the shape of the cooler is obtained from the plans of the factory in which it is installed.

As can be seen in Figure 15, the initial transformation of the extracted contour to world coordinates is just a rough approximation. The fine estimation of the projection parameters is applied next. The iterative procedure minimizes the distance between the extracted contour and the model until convergence. Finally, a nearly perfect match between the extracted contour in world coordinates and the visible part of the cooler ring is obtained. The error obtained after the iterative procedure converges is 13.36 RMS, with a mean error of 19.89 mm. This error is very low compared with the size of the rotatory cooler, with a diameter of 13 m. The result of this iterative procedure is an accurate estimation of the projection parameters that can be used to rectify the infrared images. The estimation of the projection parameters is valid while the position of the camera is not changed.

The next step is the estimation and compensation of the movement of the material in the cooler. In this case the approach used to monitor temperature is to update the position of the measurement region according to the estimated movement. Therefore, in this case movement compensation is applied as an equal movement to the position of the measurement region.

**Figure 16:**
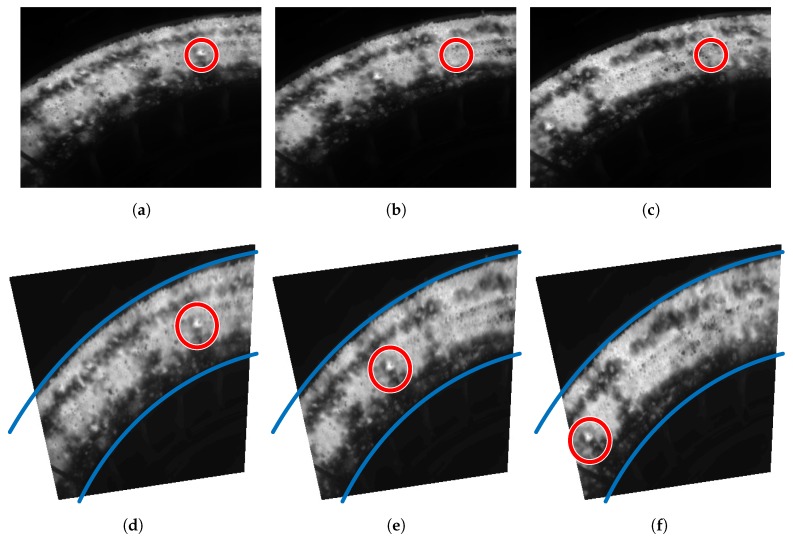
Result of the motion compensation procedure for the sinter material. (**a**) Raw image at $t_{0}$; (**b**) Raw image at $t_{1}$; (**c**) Raw image at $t_{2}$; (**d**) Rectified image at $t_{0}$; (**e**) Rectified image at $t_{1}$ with the position of the measurement region updated; (**f)** Rectified image at $t_{2}$ with the position of the measurement region updated.

Figure 16 shows the result of the estimation and compensation of the movement of the material in the cooler. The goal is to monitor the temperature of the hot spot in the images. As expected, in the raw images monitoring cannot be performed because as soon as the material moves, the position of the measurement region is incorrect, as seen in Figure 16b,c. However, applying the proposed procedure monitoring is possible since motion is estimated and compensated accurately. As seen in Figure 16d–f the position of the circular measurement region is updated correctly according to the motion of the material. Therefore, this makes the temperature monitoring of the selected region possible.

**Figure 17:**
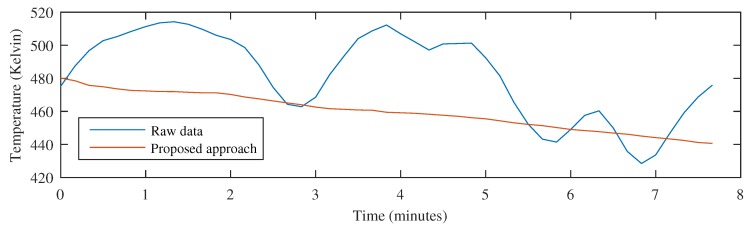
Comparing temperature monitoring using raw images and the proposed approach for the sinter material.

Figure 17 shows the result of the temperature monitoring procedure when using the raw images and the proposed procedure. The temperature values shown in this figure represent real temperature readings using a calibrated infrared camera. The signal extracted from the raw images provides information about changes in the temperature at a fixed position of the cooler. On the other hand, the signal extracted when using the proposed procedure contains an accurate representation of the temperature time history of the material at a selected region. This signal provides information about the cooling behavior and cooling per time unit, thus, it can be used to control the variables of the industrial process, including speed or air flow of the fans. Temperature monitoring at a fixed position is useless because the temperature of the material changes, and the temperature decay curve cannot be calculated. The proposed procedure solves this problem by compensating for the movement and measuring the temperature in the same area of material while it moves.

The proposed procedure not only provides the opportunity to monitor the temperature of the material as it moves, it also produces images that can be used to extract useful geometric information. For example, it is possible the measure the size of a region of interest in the image in real-world units. Also, measurement regions in the material can be established with specific sizes.

Using rectified images to estimate motion greatly simplifies the definition of the mathematical model of the movement and its estimation and compensation. However, there is another great advantage: the estimated movement is in real-world units. Therefore, it provides information that can be used to control the industrial process. Not only does the proposed procedure produce the correct temperature time history, but also the real speed of the material at any point in time.

The proposed procedure is not without limitations. It assumes that the acquisition speed of the infrared camera is much higher than the movement speed of the monitored object. This way image blurring does not affect negatively the motion estimation. Moreover, it assumes that the acquisition rate is also much higher than the speed at which the temperature of the monitored object changes.

## 4. Conclusions

This work proposes a solution for temperature monitoring when the inspected object is moved or it is affected by vibrations, very common in many scenarios. The first step is an image rectification procedure that calculates a transformed image in real-world units. This transformation produces a front-parallel projection of the image which greatly simplifies the estimation and compensation of the movements. Using this approach, motion is perfectly described using a simple 2D rigid transformation. The procedure to estimate motion proposes a robust method adapted to infrared images, but also based on well-known techniques successfully proven in visible images, such as feature detection using SURF and robust model estimation using MLESAC. The result is an accurate and robust method that provides the temperature time history of the inspected material without being affected by the movements of the material. The proposed approach assumes that the region of interest is flat, which is the case in many different types of applications.

The proposed method has been tested in laboratory and in real environments. Laboratory tests consist of a test piece that is manually moved, simulating vibrations. The proposed method robustly estimates and compensates for the simulated vibrations, providing movement free images that can be used to monitor temperature easily. The method has also been tested in a real environment: a sinter cooler. In this scenario, the material moves inside a circular ring and temperature monitoring is critical to calculate the cooling pattern and to avoid fires. The result demonstrated that the proposed work can be used to calculate the required temperature time history of the material as it moves. Moreover, additional geometric information can be extracted from result, such as the real speed of the cooler. The results of these tests validate the performance of the proposed work as a robust and accurate method to monitor the temperature of moving material. Tests are performed using long-wavelength infrared cameras, but the proposed approach could also be used with high-end mid-wavelength infrared cameras to monitor the temperature of fast moving objects.

## Figures and Tables

**Table 1:** Technical specifications of the infrared camera FLIR T450sc used in the experiments.

Camera	FLIR T450sc
Temperature range	−20 to +120 ${}^{\circ }$C
Thermal sensitivity/NETD	30 mK at 30 ${}^{\circ }$C
Detector	320 × 240 Uncooled Focal Plane Array (UFPA)
Spectral range	7.5 − 13 μm
Image frequency	60 Hz
Spatial resolution	1.36 mrad
Field of view (FOV)	25${}^{\circ }$ × 19${}^{\circ }$
Detector pitch ( μm)	25

**Table 2:** Technical specifications of the infrared camera FLIR A315 used in the experiments.

Camera	FLIR A315
Temperature range	0 to + 500 ${}^{\circ }$C
Thermal sensitivity/NETD	50 mK at 30 ${}^{\circ }$C
Detector	320 × 240 Uncooled Focal Plane Array (UFPA)
Spectral range	7.5 −14 μm
Image frequency	60 Hz
Spatial resolution	1.36 mrad
Field of view (FOV)	25${}^{\circ }$ × 18.8${}^{\circ }$
Detector pitch ( μm)	25

## References

[1] Michalski L., Eckersdorf K., Kucharski J., McGhee J. (2001). Temperature Measurement.

[2] Usamentiaga R., Venegas P., Guerediaga J., Vega L., Molleda J., Bulnes F.G. (2014). Infrared thermography for temperature measurement and non-destructive testing. Sensors.

[3] Jadin M.S., Taib S. (2012). Recent progress in diagnosing the reliability of electrical equipment by using infrared thermography. Infrared Phys. Technol..

[4] Wang H., Jiang L., Liaw P., Brooks C., Klarstrom D. (2000). Infrared temperature mapping of ULTIMET alloy during high-cycle fatigue tests. Metall. Mater. Trans. A.

[5] Maldague X. (2001). Theory and Practice of Infrared Technology for Nondestructive Testing.

[6] Hong T., Koo C., Kim J., Lee M., Jeong K. (2015). A review on sustainable construction management strategies for monitoring, diagnosing, and retrofitting the building’s dynamic energy performance: Focused on the operation and maintenance phase. Appl. Energy.

[7] Usamentiaga R., Molleda J., Garcia D.F., Bulnes F.G. (2013). Monitoring sintering burn-through point using infrared thermography. Sensors.

[8] Lahiri B., Bagavathiappan S., Jayakumar T., Philip J. (2012). Medical applications of infrared thermography: A review. Infrared Phys. Technol..

[9] Arena G., Rippa M., Mormile P., Grilli M., Paturzo M., Fatigati G., Ferraro P. Concurrent studies on artworks by digital speckle pattern interferometry and thermographic analysis. Proceedings of the SPIE OPTO International Society for Optics and Photonics.

[10] Al-doski J., Mansor S.B., Shafri H.Z.B.M. (2016). Thermal Imaging for Pests Detecting—A Review. Int. J. Agric. For. Plant..

[11] Usamentiaga R., Molleda J., García D.F., Granda J.C., Rendueles J.L. (2012). Temperature measurement of molten pig iron with slag characterization and detection using infrared computer vision. IEEE Trans. Instrum. Meas..

[12] Usamentiaga R., Molleda J., Garcia D.F. (2014). Structured-Light Sensor Using Two Laser Stripes for 3D Reconstruction without Vibrations. Sensors.

[13] Morimoto C., Chellappa R. Evaluation of image stabilization algorithms. Proceedings of the 1998 IEEE International Conference on Acoustics, Speech and Signal Processing.

[14] Ertürk S. (2002). Real-time digital image stabilization using Kalman filters. Real-Time Imaging.

[15] Ertürk S. (2003). Digital image stabilization with sub-image phase correlation based global motion estimation. IEEE Trans. Consum. Electron..

[16] Moderhak M. (2011). FFT spectra based matching algorithm for active dynamic thermography. Quant. InfraRed Thermogr. J..

[17] Hidalgo-Gato R., Mingo P., López-Higuera J.M., Madruga F.J. (2012). Pre-processing techniques of thermal sequences applied to online welding monitoring. Quant. InfraRed Thermogr. J..

[18] Croft D., Devasia S. (1999). Vibration compensation for high speed scanning tunneling microscopy. Rev. Sci. Instrum..

[19] Usamentiaga R., Garcia D., Molleda J., Bulnes F., Bonet G. (2014). Vibrations in steel strips: Effects on flatness measurement and filtering. IEEE Trans. Ind. Appl..

[20] Rajagopalan A., Chellappa R. (2014). Motion Deblurring: Algorithms and Systems.

[21] Oswald-Tranta B., Sorger M., O’Leary P. (2010). Motion deblurring of infrared images from a microbolometer camera. Infrared Phys. Technol..

[22] Usamentiaga R. (2016). Easy rectification for infrared images. Infrared Phys. Technol..

[23] Canny J. (1986). A computational approach to edge detection. IEEE Trans. Pattern Anal. Mach. Intell..

[24] Phillips J.M., Liu R., Tomasi C. Outlier robust ICP for minimizing fractional RMSD. Proceedings of the IEEE Sixth International Conference on 3-D Digital Imaging and Modeling (3DIM’07).

[25] Trucco E., Verri A. (1998). Introductory Techniques for 3-D Computer Vision.

[26] Bay H., Ess A., Tuytelaars T., Van Gool L. (2008). Speeded-up robust features (SURF). Comput. Vision Image Underst..

[27] Sezgin M., Sankur B. (2004). Survey over image thresholding techniques and quantitative performance evaluation. J. Electron. Imaging.

[28] Zuiderveld K. (1994). Contrast limited adaptive histogram equalization. Graphics Gems IV.

[29] Usamentiaga R., García D.F., Molleda J. (2015). Efficient registration of 2D points to CAD models for real-time applications. J. Real-Time Image Process..

[30] Torr P.H., Zisserman A. (2000). MLESAC: A new robust estimator with application to estimating image geometry. Comput. Vision Image Underst..

[31] Fischler M.A., Bolles R.C. (1981). Random sample consensus: A paradigm for model fitting with applications to image analysis and automated cartography. Commun. ACM.

[32] ISO 18434-1:2008 (2011). Condition Monitoring and Diagnostics of Machines —Thermography—Part 1: General Procedures.

[33] Kang S.J.L. (2004). Sintering: Densification, Grain Growth and Microstructure.

